# Pilot study of Myocardial MIBG Scintigraphy with 123I‐FP‐CIT SPECT in Isolated REM Sleep Behavior Disorder

**DOI:** 10.1002/alz.091810

**Published:** 2025-01-09

**Authors:** Toji Miyagawa, Cynthia Vernon, Scott A. Przybelski, Hoon‐Ki Min, Julie A. Fields, Leah K. Forsberg, Erik K St. Louis, Kejal Kantarci, Val J. Lowe, Brad F. Boeve

**Affiliations:** ^1^ Mayo Clinic, Rochester, MN USA

## Abstract

**Background:**

Autopsy studies in Lewy Body Disease (LBD) indicate that cardiac sympathetic denervation precedes Lewy body pathology and neuronal loss in the brain. Myocardial 123I‐metaiodobenzylguanidine (MIBG) scintigraphy noninvasively assesses postganglionic cardiac sympathetic denervation in LBD and is considered an important biomarker in the international diagnostic criteria of Dementia with Lewy Bodies and Parkinson’s Disease (PD). Despite the internationally recognized importance of MIBG scintigraphy in LBD, its use in neurodegenerative disorders is not FDA approved for this indication and is rarely used in the US for neurological research. We sought to add MIBG scintigraphy to our multimodal imaging research in prodromal LBD. We launched pilot MIBG scintigraphy study in isolated REM Sleep Behavior Disorder (iRBD) individuals, who are typically regarded as representing evolving LBD.

**Method:**

We conducted MIBG scintigraphy and 123I‐FP‐CIT SPECT in seven individuals with iRBD including two RBD with mild cognitive impairment (MCI), one with PD, and one healthy control (mean age of 71.5±8.3). Heart‐to‐mediastinum (H/M) ratio of the MIBG uptake on delayed image (3‐4 hours after Adreview® injection) was calculated with MIM. Cutoff value of 1.68 was used for determining abnormal H/M ratio (Yoshita et al, 2006). Nigrostriatal dopamine transporter uptake was evaluated with DaTQUANT 2.0 software.

**Result:**

Four iRBD participants including two RBD with MCI had abnormal MIBG scintigraphy (mean age 73.5±8.6, delayed H/M ratio 1.25±0.29)) and three iRBD participants had a normal scan (mean age 67.8±4.5, delayed H/M ratio 2.57±0.25). Delayed H/M ratio was 1.00 for the PD participant and 2.60 for healthy control participant. Among the four iRBD participants with abnormal MIBG scintigraphy, one participant had 123I‐FP‐CIT SPECT with DaTQUANT putamen z‐score <‐2.0 and three participants had z‐score in the range of ‐1.5 and ‐1.0. All iRBD participants with normal MIBG scintigraphy had normal 123I‐FP‐CIT SPECT with DaTQUANT putamen z‐score >‐1.0.

**Conclusion:**

MIBG scintigraphy was abnormal in half of this small cohort. Our preliminary data suggests its utility in detecting early signs of evolving LBD. Increasing the number of the MIBG scintigraphy in iRBD participants and controls are planned to determine if MIBG scintigraphy can facilitate early detection of progression for use in clinical trials targeting LBD.

